# Obesity, perceived weight discrimination, and psychological well‐being in older adults in England

**DOI:** 10.1002/oby.21052

**Published:** 2015-03-25

**Authors:** Sarah E. Jackson, Rebecca J. Beeken, Jane Wardle

**Affiliations:** ^1^ Health Behaviour Research CentreDepartment of Epidemiology and Public HealthUniversity College LondonLondonUK

## Abstract

**Objective:**

To examine whether the adverse effect of obesity on psychological well‐being can be explained by weight discrimination.

**Methods:**

The study sample included 5056 older (≥50 y) men and women living in England and participating in the English Longitudinal Study of Ageing. Participants reported experiences of weight discrimination in everyday life and completed measures of quality of life (CASP‐19 scale), life satisfaction (Satisfaction With Life Scale), and depressive symptoms (eight‐item CES‐D scale). Height and weight were objectively measured, with obesity defined as BMI ≥30 kg/m^2^. Mediation analyses were used to test the role of perceived weight discrimination in the relationship between obesity and each psychological factor.

**Results:**

Obesity, weight discrimination, and psychological well‐being were all significantly inter‐related. Mediation models revealed significant indirect effects of obesity through perceived weight discrimination on quality of life (β = −0.072, SE = 0.008), life satisfaction (β = −0.038, SE = 0.008), and depressive symptoms (β = 0.057, SE = 0.008), with perceived weight discrimination explaining approximately 40% (range: 39.5‐44.1%) of the total association between obesity and psychological well‐being.

**Conclusions:**

Perceived weight discrimination explains a substantial proportion of the association between obesity and psychological well‐being in English older adults. Efforts to reduce weight stigma in society could help to reduce the psychological burden of obesity.

## Introduction

In addition to the well‐documented physical health risks associated with obesity 1, adverse effects on psychological well‐being have long been recognized. In 1985, the National Institutes of Health drew attention to the “enormous psychological burden” created by obesity 2. This may to some extent overstate the case, but there is certainly evidence that individuals with obesity experience poorer quality of life 3, body image disturbance 4, and lower self‐esteem 5 and are at increased risk of depression and other psychiatric disorders 6, 7. Effects are typically strongest among people who are very obese, with one study finding that many patients awaiting bariatric surgery would prefer to be normal weight with a major handicap (deaf/blind/one leg amputated) than stay morbidly obese, and the majority saying they would rather be normal weight than a morbidly obese multimillionaire 8.

Weight stigma is often cited as a potential mechanism leading from obesity to poorer psychological well‐being 4, 5, 7, 9. Prejudice against individuals with obesity is pervasive and rarely challenged in Western society 10. As a result, many individuals with obesity, and particularly those with severe obesity, report being discriminated against because of their weight in their everyday lives 11, 12. Given that weight stigma and discrimination have both been shown to have a negative impact on psychological health outcomes, including well‐being 10, depression 13, 14, self‐esteem and self‐acceptance 13, 15, and body image dissatisfaction 13, 16, this might explain why people with obesity suffer psychologically.

Only one study to our knowledge has tested the mediating effect of weight‐related discrimination, showing a significant reduction in the association between obesity and self‐acceptance after adjusting for perceived weight discrimination 15. None have examined the role of discrimination in relation to more global indices of psychological well‐being, such as quality of life or depression. The aim of the present study was therefore to investigate the extent to which perceived weight discrimination mediates associations between obesity and three markers of well‐being: quality of life, life satisfaction, and depressive symptoms.

## Methods

### Study population

Data were from the English Longitudinal Study of Ageing (ELSA); a cohort study of older adults (≥50 y) living in England 17. ELSA participants were recruited from an annual cross‐sectional survey of households, and comparisons of their socio‐demographic characteristics against the national census indicate that the sample is broadly representative of the older English population 17. Six waves of ELSA data have been collected to date, starting in 2002 and repeated every other year since. At each assessment, participants complete an interview and questionnaires, and in alternate (even) waves nurse visits are conducted to obtain objective measures of health status, including body weight. Discrimination was assessed in wave 5 (2010‐2011). The present analyses used anthropometric data from wave 4 (2008‐2009) and data on discrimination and psychological well‐being from wave 5. Our sample included participants with complete data on discrimination, BMI, and at least one psychological outcome (*n* = 5056).^1^ Participants gave full informed consent and ethical approval was obtained from the London Multi‐Centre Research Ethics Committee.

### Measures

#### Obesity

Weight was measured by nurses to the nearest 0.1 kg using portable electronic scales, and height was measured to the nearest millimeter using a portable stadiometer. Nurses recorded any factors that might have compromised the reliability of the measurements (e.g., participant was stooped/unwilling to remove shoes) and these cases were excluded. Underweight was defined as a BMI <18.5, normal weight as BMI 18.5‐25, overweight as BMI 25‐29.9, and obesity as BMI ≥30.

#### Weight discrimination

Questions on perceived discrimination were based on items developed and used widely in other longitudinal studies in the USA 12, 15, 19. Participants were asked about the frequency of five discriminatory experiences: “*In your day‐to‐day life, how often have any of the following things happened to you: (*1*) you are treated with less respect or courtesy; (*2*) you receive poorer service than other people in restaurants and stores; (*3*) people act as if they think you are not clever; (*4*) you are threatened or harassed; and (*5*) you receive poorer service or treatment than other people from doctors or hospitals (almost every day/at least once a week/a few times a month/a few times a year/less than once a year/never)*.” Because data were skewed, with most participants reporting never experiencing discrimination, we dichotomized responses to indicate whether or not respondents had ever experienced discrimination (never vs. all other options). A follow‐up question asked participants to indicate the reason(s) for discrimination from a list including weight, age, gender, race, and physical disability. For the purpose of these analyses, perceived weight discrimination was defined as experiencing discrimination and attributing it to weight.

#### Psychological well‐being

We included three measures of psychological well‐being in our analyses: quality of life, life satisfaction, and depressive symptoms. Our rationale for including these distinct constructs was to have one broad measure of well‐being, one of positive affect, and one of negative affect.

Quality of life was assessed with the CASP‐19 20, a scale designed to measure quality of life in older people. Items cover four domains: control (e.g., “*I feel that what happens to me is out of my control”*), autonomy (e.g., “*My health stops me from doing things I want to do”*), self‐realization (e.g., “*I feel that life is full of opportunities”*), and pleasure (e.g., “*I enjoy being in the company of others”*). Respondents were asked how often each statement applies to them (often = 0, sometimes = 1, not often = 2, never = 3). Positively worded items were reverse scored so that higher scores indicated higher quality of life. The Cronbach α in the present sample was 0.86.

Life satisfaction was assessed with the Satisfaction With Life Scale (SWLS) 21, which asks the extent to which participants agree with five statements (e.g., “*In most ways my life is close to my ideal”*). Responses were on a Likert scale from 0 (strongly disagree) to 6 (strongly agree). The Cronbach α was 0.91.

Depressive symptoms were assessed with an eight‐item version of the Center for Epidemiologic Studies Depression Scale (CES‐D) 22. This asks about feelings over the last week (e.g., “*Over the last week have you felt sad”*), with binary response options (1 = yes, 0 = no). Positively framed items were reverse scored. The eight‐item version has comparable validity and reliability to the original 20‐item CES‐D 23. The Cronbach α was 0.77.

For each of these three scales, we computed mean scores for participants with data on at least 75% of items to maximize the number of participants that could be included in the analyses. Standardized scores (*z*‐scores) were calculated for each scale for ease of comparison across the three scales.

#### Demographic information

Interviewers collected information on age, sex, ethnicity, and household nonpension wealth. Because of the small number of participants from non‐white ethnic groups, we categorized ethnicity as white vs. non‐white. Wealth was categorized into five equal groups of net total nonpension wealth measured at benefit unit level (a benefit unit is a couple or single person along with any dependent children they might have) across all ELSA participants who took part in wave 5. Wealth has been identified as a particularly appropriate indicator of SES in this age group 24.

### Statistical analysis

All analyses were conducted using STATA version 13.1 (STATA Corporation, TX, USA). Age, sex, and wealth (as a proxy for SES) were entered as covariates for all the analyses because of their known associations with obesity 25 and psychological well‐being (e.g., 26,27). Ethnicity was not adjusted for because participants were almost exclusively white (98%). We used logistic regression to test the association between obesity and perceived weight discrimination, and analyses of covariance to test for differences in psychological well‐being by obesity and perceived weight discrimination.

**Figure 1:**
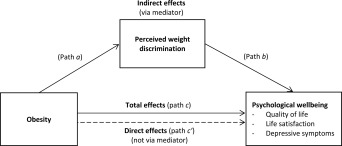
Mediation model of associations between obesity and psychological wellbeing via perceived weight discrimination.

Mediation analyses were used to test the hypothesis that perceived weight discrimination mediated the relationship between obesity and psychological well‐being (Figure 1). We calculated total, direct, and indirect effects, and tested the significance of the indirect effect using the Sobel test 28, 29. The total effect (path *c*) of an independent variable (IV) on a dependent variable (DV) consists of a direct effect (path *c*′) of the IV on the DV and an indirect effect (path *a* × *b*) of the IV on the DV via a proposed mediator. Path *a* represents the effect of the IV on the mediator, and path *b* is the effect of the mediator on the DV. In these analyses, obesity was the IV, psychological variables were the DVs, and perceived weight discrimination was the mediator. Standardized scores were used for indices of psychological well‐being for ease of comparison across the three psychological variables. We used bootstrapping with 5000 sampling replications to estimate the 95% confidence interval (CI) 30. Bootstrap tests of mediation are considered a better method of testing the significance of indirect effects than the Sobel test because they do not assume a normal distribution and therefore reduce the likelihood of type 2 error 30, 31. If the 95% CI does not include 0 the indirect effect is considered significant 30. We also calculated effect ratios that reflect the proportion of the total effect of the IV on the DV that is explained by the mediator; in this case, the proportion of the total effect of obesity on psychological well‐being that is explained by perceived weight discrimination. For example, an effect ratio of 0.5 would indicate that half of the total effect is explained by the mediator 31. In addition to comparing all obese individuals with those who were not obese, we repeated these mediation models separately for those with class I obesity (BMI 30‐34.9) and class II/III obesity (BMI ≥35), to examine whether the “average” effects were driven by participants in the more severely obese group. Previous research has indicated that the prevalence of weight discrimination increases substantially above a BMI of 35 11, and psychological impairment is greater with more severe obesity 18.

**Figure 2:**
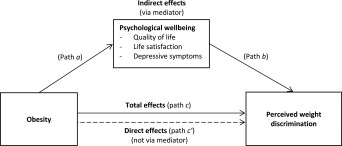
Mediation model of associations between obesity and perceived weight discrimination via psychological well‐being.

We performed sensitivity analyses testing for mediation of the association between obesity and psychological well‐being by two other types of perceived discrimination (age and sex discrimination; the most prevalent forms of perceived discrimination reported by the sample, at 40% and 11% respectively) in order to establish whether effects were—as predicted—specific to weight discrimination or applied to experiences of discrimination in general. Additionally, because previous research has indicated that there may also be causal effects in the other direction [i.e., lower psychological well‐being is associated with greater likelihood of perceiving discrimination 32], we also tested this model. We used multiple mediation analysis 33 with obesity as the IV, weight discrimination as the DV, and quality of life, life satisfaction, and depressive symptoms as mediators (Figure 2). We followed the product‐of‐coefficients method using seemingly unrelated regression and bootstrapping with 5000 replications.

## Results

Characteristics of the study sample are shown in Table 1. Participants were on average 67.5 years old, 55.9% were women, and 97.9% were white. Mean BMI was 28.2, and 31.2% of participants were obese. Individuals with obesity were on average younger (66.8 vs. 67.8 y, *P* < 0.001) and less wealthy (*P* < 0.001) than nonobese individuals, and a greater proportion were female (60.4% vs. 53.8%, *P* < 0.001). Ethnicity did not differ by obesity status (*P* = 0.327). Weight discrimination was reported by 4.6% but was strongly related to weight status, with 12.9% of individuals with obesity reporting weight discrimination (6.7% of class I obese, 26.8% of class II/III obese) and only 0.9% of nonobese individuals (2.6% of underweight, 0.7% of normal weight, 0.9% of overweight; adjusted OR [obese vs. nonobese] = 15.18, 95% CI = 10.26 to 22.48, *P* < 0.001).

**Table 1:** Characteristics of the study sample (*n* = 5056) – mean ± SD or % (*n*)

	Whole sample (*n* = 5056)	Nonobese (*n* = 3480)	Obese (*n* = 1576)	*P*
**Age (y)**	67.46 ± 8.85	67.80 ± 9.02	66.76 ± 8.43	<0.001
**Sex**				
**Male**	44.1 (2231)	46.2 (1607)	39.6 (624)	<0.001
**Female**	55.9 (2825)	53.8 (1873)	60.4 (952)	‐
**Ethnicity**				
**White**	97.9 (4949)	98.0 (3411)	97.6 (1538)	0.327
**Non‐white**	2.1 (107)	2.0 (69)	2.4 (38)	‐
**Wealth quintile** a				
**1 (lowest)**	15.8 (798)	13.4 (468)	20.9 (330)	<0.001
**2**	19.4 (979)	17.9 (624)	22.5 (355)	‐
**3**	19.9 (1005)	19.1 (664)	21.6 (341)	‐
**4**	21.8 (1103)	23.1 (804)	19.0 (299)	‐
**5 (highest)**	23.2 (1171)	26.4 (920)	15.9 (251)	‐
**Weight (kg)**	77.68 ± 15.77	70.98 ± 11.53	92.48 ± 13.66	<0.001
**BMI (kg/m^2^)**	28.19 ± 5.05	25.52 ± 2.73	34.10 ± 3.84	<0.001
**Weight status**				
**Underweight**	0.8 (38)	1.1 (38)	‐	‐
**Normal weight**	26.6 (1344)	38.6 (1344)	‐	‐
**Overweight**	41.5 (2098)	60.3 (2098)	‐	‐
**Obese**	31.2 (1576)	‐	100 (1576)	‐
**Class I obese**	21.6 (1091)	‐	69.2 (1091)	‐
**Class II/III obese**	9.6 (485)	‐	30.8 (485)	‐
**Perceived weight discrimination**	4.6 (233)	0.9 (30)	12.9 (203)	<0.001
**Quality of life**				<0.001
**Mean score**	2.16 ± 0.46	2.20 ± 0.45	2.08 ± 0.47	<0.001
***z*‐score**	0.00 ± 1.00	0.08 ± 0.98	−0.18 ± 1.02	‐
**Life satisfaction**				<0.001
**Mean score**	4.14 ± 1.27	4.20 ± 1.23	4.00 ± 1.33	<0.001
***z*‐score**	0.00 ± 1.00	0.05 ± 0.97	−0.11 ± 1.05	‐
**Depressive symptoms**				<0.001
**Mean score**	0.18 ± 0.24	0.16 ± 0.23	0.21 ± 0.25	<0.001
***z*‐score**	0.00 ± 1.00	−0.07 ± 0.96	0.03 ± 1.06	‐

a Weight quintiles were derived from the whole ELSA sample. *P* values are for the difference between nonobese and obese individuals.

Obesity was significantly related to psychological well‐being, although effect sizes were modest. Individuals with obesity reported lower quality of life (*P* < 0.001), lower life satisfaction (*P* = 0.005), and more depressive symptoms (*P* < 0.001) than those without obesity (Table 2). Among the obese group, psychological impairment was greater in those with class II/III obesity than those with class I obesity (quality of life: *P* < 0.001; life satisfaction: *P* = 0.004; depressive symptoms: *P* < 0.001). Individuals who reported experiences of weight discrimination also had poorer psychological well‐being in all three domains than those who did not report weight discrimination (*P* < 0.001) (Table 2). Associations between weight discrimination and psychological well‐being were similar in participants who were excluded for missing weight data to those in the included cases.

**Table 2:** Mean ± SE psychological well‐being by obesity status and perceived weight discrimination

	Obesity				Perceived weight discrimination			
	No	Yes	*F*	*P*	No	Yes	*F*	*P*
**Quality of life**								
**Mean score**	2.19 ± 0.01	2.11 ± 0.11	38.95	<0.001	2.18 ± 0.01	1.88 ± 0.03	90.42	<0.001
***z*‐score**	0.06 ± 0.02	−0.13 ± 0.02	‐	‐	0.03 ± 0.01	−0.61 ± 0.07	‐	‐
**Life satisfaction**								
**Mean score**	4.17 ± 0.02	4.06 ± 0.03	8.07	0.005	4.16 ± 0.02	3.70 ± 0.09	26.04	<0.001
***z*‐score**	0.03 ± 0.02	−0.06 ± 0.03	‐	‐	0.02 ± 0.01	−0.34 ± 0.07	‐	‐
**Depressive symptoms**								
**Mean score**	0.17 ± 0.004	0.20 ± 0.006	21.88	<0.001	0.17 ± 0.003	0.29 ± 0.02	49.57	<0.001
***z*‐score**	−0.04 ± 0.02	0.10 ± 0.02	‐	‐	−0.02 ± 0.01	0.46 ± 0.07	‐	‐

Values are adjusted for BMI, age, sex, and wealth.

SE = standard error.

Table 3 summarizes the results of the mediation analyses (path *c*, path *c'*, and indirect effects in Figure 1). We observed significant indirect effects of obesity through weight discrimination on all three measures of psychological well‐being (quality of life: β = −0.072, SE = 0.008, 95% CI = −0.091 to −0.054; life satisfaction: β = −0.038, SE = 0.008, 95% CI = −0.058 to −0.019; depressive symptoms: β = 0.057, SE = 0.008, 95% CI = 0.036 to 0.078). Effect ratios indicated that weight discrimination explained just over 40% of the total effect of obesity on psychological well‐being (range: 39.5‐44.1%). There were also direct effects of obesity on quality of life (β = −0.110, SE = 0.030) and depressive symptoms (β = 0.081, SE = 0.031), but the direct effect on life satisfaction was not significant. Analysis of associations between obesity and the four domains of quality of life revealed consistent evidence of mediation by weight discrimination, with effect ratios ranging from 31.0% (autonomy) to 46.4% (pleasure) (Supporting Information Table 1). Despite higher prevalence of perceived weight discrimination and greater psychological impairment among individuals with more severe (class II/III) obesity than those with class I obesity, we observed no notable differences in the mediating effect of perceived weight discrimination when we ran mediation analyses separately for the two obese groups (data not shown).

**Table 3:** Models testing mediation of associations between obesity and psychological well‐being by perceived weight discrimination (see Figure 1)

	Coeff.	SE	*P* a	Bootstrap 95% CI	Effect ratio
**Obesity and quality of life**					
**Total effect (path *c*)**	−0.182	0.029	<0.001	‐	‐
**Direct effect (path *c*')**	−0.110	0.030	<0.001	‐	‐
**Indirect effect (via mediator)**	−0.072	0.008	<0.001	[−0.091; −0.054]	0.395
**Obesity and life satisfaction**					
**Total effect (path *c*)**	−0.086	0.030	0.004	‐	‐
**Direct effect (path *c*')**	−0.048	0.031	0.123	‐	‐
**Indirect effect (via mediator)**	−0.038	0.008	<0.001	[−0.058; −0.019]	0.441
**Obesity and depressive symptoms**					
**Total effect (path *c*)**	0.137	0.030	<0.001	‐	‐
**Direct effect (path *c*')**	0.081	0.031	0.009	‐	‐
**Indirect effect (via mediator)**	0.057	0.008	<0.001	[0.036; 0.078]	0.412

Models use *z*‐scores for all psychological well‐being variables.

All models are adjusted for age, sex, and wealth.

Coeff. = coefficient; SE = standard error; CI = confidence interval.

a *P* values shown for indirect effects are derived from the Sobel test for consistency with total and direct effects; however, bootstrap 95% confidence intervals provide a more robust indication of significant mediation (see Methods for more details).

We repeated the mediation analyses substituting age discrimination and sex discrimination in turn for weight discrimination to investigate whether mediation was specific to weight discrimination (Supporting Information Table 2). Although perceived discrimination on the basis of age or sex was significantly associated with poorer psychological well‐being, we observed no evidence of mediation of the effect of obesity.

We also tested the reverse model (Figure 2) to investigate whether reports of weight discrimination by individuals with obesity could be explained by their lower well‐being (Table 4). We observed both a direct effect of obesity on perceived weight discrimination (β = 0.106, SE = 0.006) and a small indirect effect of psychological well‐being (β for total indirect effect = 0.006, SE = 0.001, 95% CI = 0.004 to 0.009), driven predominantly by a mediating effect of quality of life (β = 0.006, SE = 0.001, 95% CI = 0.004 to 0.009). Psychological variables explained 5.6% of the association between obesity and perceived weight discrimination.

**Table 4:** Model testing mediation of the association between obesity and perceived weight discrimination by psychological well‐being (see Figure 2)

	Coeff.	SE	*P* a	Bootstrap 95% CI	Effect ratio
**Total effect (path *c*)**	0.112	0.006	<0.001	‐	‐
**Direct effect (path *c*')**	0.106	0.006	<0.001	‐	‐
**Indirect effect (via mediators)**	0.0063	0.001	<0.001	[0.004; 0.009]	0.056
**Indirect effect (via quality of life)**	0.0059	0.001	<0.001	[0.004; 0.009]	0.053
**Indirect effect (via life satisfaction)**	−0.0009	0.0005	0.072	[−0.002; −0.0001]	−0.008
**Indirect effect (via depressive symptoms)**	0.0013	0.0007	0.067	[0.0001; 0.003]	0.012

Model uses *z*‐scores for all psychological well‐being variables.

Model is adjusted for age, sex, and wealth.

Coeff. = coefficient; SE = standard error; CI = confidence interval.

a *P* values shown for indirect effects are derived from the Sobel test for consistency with total and direct effects; however, bootstrap 95% confidence intervals provide a more robust indication of significant mediation (see Methods for more details).

## Discussion

In this study, we examined associations between obesity, perceived weight discrimination, and three markers of psychological well‐being: quality of life, life satisfaction, and depressive symptoms. Individuals with obesity showed poorer well‐being in all three domains, although effect sizes were modest. They were also substantially more likely to report weight discrimination. We used mediation models with bootstrapping to test the proposition that associations between obesity and well‐being are mediated by weight discrimination and found that approximately 40% of the total effect of obesity on psychological well‐being could be explained by perceptions of weight discrimination.

In order to rule out the possibility that any discrimination would have the same effect—i.e., it was nothing to do with weight *per se*, we carried out sensitivity analyses using other types of discrimination. Although age and sex discrimination were commonly reported, and had negative effects on well‐being, they did not explain the lower levels of well‐being among participants with obesity compared to those without obesity. This finding is consistent with a previous study that showed that the relationship between obesity and self‐acceptance was not attenuated when general experiences of discrimination were adjusted for, but became nonsignificant in analyses controlling for appearance‐related discrimination 15.

The fact that perceived weight discrimination explained such a significant proportion of the unique variance in the association between obesity and psychological well‐being emphasizes the need to combat weight stigmatization in society. Public health campaigns designed to tackle obesity may inadvertently stigmatize individuals with obesity, with messages that emphasize volitional control of body weight and minimize the importance of nonvolitional factors that contribute to obesity 34. There have been calls for such interventions to focus on facilitating behavioral change and to endorse health rather than “ideal weight” as the primary desired outcome 34. Other suggested strategies to reduce weight bias in the public health context include training health professionals about stigma and stereotyping, involving people with obesity in finding solutions to stigmatizing programs and policies, and ensuring consistent implementation of nonstigmatizing messages and approaches 35. Promoting self‐acceptance for individuals with obesity could also help to minimize the impact of perceived discrimination and improve well‐being. In individuals with obesity who had recently completed a weight loss program, a brief acceptance‐based intervention that focused on weight‐related stigmatizing thoughts was associated with significant improvements in psychological distress and quality of life 36. However, directly addressing the issue of weight discrimination, rather than simply teaching people with obesity how best to cope with it, will inevitably have a greater impact on well‐being.

The occurrence of discrimination is difficult to determine objectively because it relies on interpretation of the intentions of others. As such, discrimination can occur without being perceived by the individual who is discriminated against, and equally, it can be perceived in cases where it did not occur. In the latter situation, a person's psychological state may influence the way they interpret others' behavior and hence whether discrimination is perceived. A study exploring perceptions of race discrimination in minority adolescents in the US showed that those with higher levels of depression or anxiety were more likely to perceive discrimination 32. In the present study, we tested for this “reverse effect” and observed small but significant indirect effects of quality of life, life satisfaction, and depressive symptoms on perceived weight discrimination, suggesting that differences in these psychological factors contribute to individuals with obesity being more likely than those without obesity to perceive weight discrimination. However, the total indirect effect of psychological well‐being explained only 6% of the association between obesity and perceived weight discrimination, indicating that there are other important factors that account for this relationship.

This study had a number of strengths. It used a large sample drawn from a nationally representative cohort, in which the prevalence of perceived weight discrimination was comparable to previous estimates in the equivalent age group in the US population 37. Many studies on weight discrimination or psychological well‐being have been limited to smaller, treatment‐seeking obese samples for whom weight discrimination may have been part of the motivation to seek treatment. These groups may not be representative of individuals with obesity in the general population who tend to suffer less psychological disturbance and are typically less obese, so may be less likely to perceive weight discrimination 5, 38, 39. The question on discrimination was phrased generally at first and then weight was included among a list of other possible attributions for discrimination, limiting reporting bias among obese respondents. The availability of objective measurements of height and weight in ELSA is also an advantage because many large longitudinal studies rely on self‐reported data.

However, there were also limitations. Weight was not measured in the same data collection wave as discrimination, and participants may have changed weight status prior to reporting discrimination. We did not have complete data on weight and it is possible that people most troubled by their weight were more likely to refuse to be weighed. However, although missing cases inevitably pose a source of bias, we think it unlikely that exclusion of ELSA respondents with missing weight data would have resulted in substantial over‐ or under‐estimation of the mediation effect because the discrimination‐well‐being association was the same in that group as in the total included group. Weight discrimination was self‐reported and was therefore subject to recall bias and only reflected participants' own perceptions of discrimination. These results therefore estimate the impact of believing that one has been a target of weight discrimination as opposed to the impact of weight discrimination *per se*. It is possible that the timing of discriminatory experiences was years prior to when they were reported, which would make mediation effects less plausible as the purported mediator would be temporally prior to the purported IV. The sample comprising older adults may have implications, as high BMI over the life course is a predictor of premature mortality 40; thus the individuals with obesity in this sample may be positively selected on some trait that both helped them to stay alive and remain healthy enough to participate in a major data collection effort. In addition, as the sample was predominantly white (98%) results may not generalize to other ethnic groups. Finally, our analyses were restricted to participants with data on discrimination, BMI, and at least one psychological outcome; and although participants in the analyzed sample matched the total ELSA sample at wave 5 on age and sex, they were on average slightly wealthier and heavier, so results may not be population‐representative.

Future research could extend our findings by investigating the extent to which weight discrimination mediates associations between obesity and other measures of well‐being, such as self‐esteem. It would also be interesting to examine differences in associations between obesity, weight discrimination, and well‐being across demographic subgroups. The greater social pressures on women than men to maintain a slender physique may mean women with obesity are more vulnerable to the adverse psychological effects of weight discrimination, so the mediating effect may be stronger in women than men. Likewise, it is possible that weight discrimination explains a larger proportion of the association between obesity and well‐being in younger populations, where obesity‐associated health problems, which may lead to poorer well‐being among individuals with obesity, have had less time to develop.

In summary, our results indicate that a substantial proportion of the association between obesity and psychological well‐being can be explained by perceptions of weight discrimination. Concerted efforts to reduce weight stigma in society could therefore help to alleviate the psychological burden of obesity.

## Supporting information

Supporting Information

## Footnotes

1 The prevalence of weight discrimination was slightly higher in participants for whom weight data were missing than in the analyzed sample (6.9% vs. 4.6%).
