# Supplementary material for: Obesity, perceived weight discrimination, and psychological well‐being in older adults in England
Source: Obesity (Silver Spring). 2015 Mar 25;23(5):1105–11. doi: 10.1002/oby.21052 (PMC4414736; doi:10.1002/oby.21052)
Supplement: Supplementary file 1 — Supporting Information [file OBY-23-1105-s001.docx]

**Obesity, perceived weight discrimination, and psychological wellbeing in older adults in England: supporting information**

**Sarah E Jackson,^1^ Rebecca J Beeken,^1^ Jane Wardle^1^**

^1^ Health Behaviour Research Centre, Department of Epidemiology and Public Health, University College London, London, UK

Tel: +44 (0)20 7679 5634. Fax: +44 (0)20 7679 8354. Email: s.e.jackson@ucl.ac.uk.

| **Supplementary Table 1** Models testing mediation of associations between obesity and domains of quality of life by perceived weight discrimination | | | | | | |
| --- | --- | --- | --- | --- | --- | --- |
|  | | **Coeff.** | **SE** | ***p**** | **Bootstrap 95% CI** | **Effect ratio** |
| Obesity and control | |  |  |  |  |  |
|  | Total effect (path *c*) | -0.154 | 0.030 | <.001 | - | - |
|  | Direct effect (path *c*’) | -0.086 | 0.031 | .005 | - | - |
|  | Indirect effect (via mediator) | -0.068 | 0.008 | <.001 | [-0.085; -0.051] | 0.441 |
| Obesity and autonomy | |  |  |  |  |  |
|  | Total effect (path *c*) | -0.227 | 0.029 | <.001 | - | - |
|  | Direct effect (path *c*’) | -0.157 | 0.030 | <.001 | - | - |
|  | Indirect effect (via mediator) | -0.070 | 0.008 | <.001 | [-0.090; -0.052] | 0.310 |
| Obesity and self-realisation | |  |  |  |  |  |
|  | Total effect (path *c*) | -0.099 | 0.029 | .001 | - | - |
|  | Direct effect (path *c*’) | -0.056 | 0.030 | .065 | - | - |
|  | Indirect effect (via mediator) | -0.043 | 0.008 | <.001 | [-0.060; -0.026] | 0.432 |
| Obesity and pleasure | |  |  |  |  |  |
|  | Total effect (path *c*) | -0.152 | 0.030 | <.001 | - | - |
|  | Direct effect (path *c*’) | -0.081 | 0.031 | .008 | - | - |
|  | Indirect effect (via mediator) | -0.071 | 0.009 | <.001 | [-0.092; -0.050] | 0.464 |
| Models use z-scores for all quality of life variables.  All models are adjusted for age, sex, and wealth.  Coeff. = coefficient; SE = standard error; CI = confidence interval.  **p* values shown for indirect effects are derived from the Sobel test for consistency with total and direct effects, however bootstrap 95% confidence intervals provide a more robust indication of significant mediation (see Method for more details). | | | | | | |

| **Supplementary Table 2**  Models testing mediation of associations between obesity and psychological wellbeing by perceived age and sex discrimination | | | | | | | |
| --- | --- | --- | --- | --- | --- | --- | --- |
|  |  | | **Coeff.** | **SE** | ***p**** | **Bootstrap 95% CI** | **Effect ratio** |
| **Age discrimination** | | |  |  |  |  |  |
|  | Quality of life | |  |  |  |  |  |
|  |  | Total effect (path *c*) | -0.182 | 0.029 | <.001 | - | - |
|  |  | Direct effect (path *c*’) | -1.173 | 0.029 | <.001 | - | - |
|  |  | Indirect effect (via mediator) | -0.009 | 0.005 | .062 | [-0.019; 0.001] | 0.051 |
|  | Life satisfaction | |  |  |  |  |  |
|  |  | Total effect (path *c*) | -0.086 | 0.030 | .004 | - | - |
|  |  | Direct effect (path *c*’) | -0.081 | 0.030 | .007 | - | - |
|  |  | Indirect effect (via mediator) | -0.005 | 0.003 | .085 | [-0.010; 0.001] | 0.055 |
|  | Depressive symptoms | |  |  |  |  |  |
|  |  | Total effect (path *c*) | 0.137 | 0.030 | <.001 | - | - |
|  |  | Direct effect (path *c*’) | 0.132 | 0.030 | <.001 | - | - |
|  |  | Indirect effect (via mediator) | 0.005 | 0.003 | .071 | [-0.0004; 0.012] | 0.039 |
| **Sex discrimination** | | |  |  |  |  |  |
|  | Quality of life | |  |  |  |  |  |
|  |  | Total effect (path *c*) | -0.182 | 0.029 | <.001 | - | - |
|  |  | Direct effect (path *c*’) | -0.181 | 0.029 | <.001 | - | - |
|  |  | Indirect effect (via mediator) | -0.001 | 0.001 | .417 | [-0.004; 0.002] | 0.006 |
|  | Life satisfaction | |  |  |  |  |  |
|  |  | Total effect (path *c*) | -0.086 | 0.030 | .004 | - | - |
|  |  | Direct effect (path *c*’) | -0.086 | 0.030 | .005 | - | - |
|  |  | Indirect effect (via mediator) | -0.0001 | 0.0003 | .815 | [-0.001; 0.001] | 0.001 |
|  | Depressive symptoms | |  |  |  |  |  |
|  |  | Total effect (path *c*) | 0.137 | 0.030 | <.001 | - | - |
|  |  | Direct effect (path *c*’) | 0.136 | 0.030 | <.001 | - | - |
|  |  | Indirect effect (via mediator) | 0.001 | 0.001 | .462 | [-0.001; 0.003] | 0.006 |
| Models use z-scores for all psychological wellbeing variables.  All models are adjusted for age, sex, and wealth.  Coeff. = coefficient; SE = standard error; CI = confidence interval.  **p* values shown for indirect effects are derived from the Sobel test for consistency with total and direct effects, however bootstrap 95% confidence intervals provide a more robust indication of significant mediation (see Method for more details). | | | | | | | |
